# Associations of intercellular adhesion molecule‐1 rs5498 polymorphism with ischemic stroke: A meta‐analysis

**DOI:** 10.1002/mgg3.643

**Published:** 2019-04-17

**Authors:** Hua Gao, Xinhua Zhang

**Affiliations:** ^1^ Department of Neurology Shanghai Sixth People's Hospital East Affiliated to Shanghai University of Medicine & Health Sciences Shanghai China; ^2^ Department of General Practice Shanghai Pudong New District Dongming Community Healthcare Center Shanghai China

**Keywords:** intercellular adhesion molecule‐1 (*ICAM‐1*), ischemic stroke (IS), meta‐analysis, polymorphisms

## Abstract

**Background:**

Recently, associations between intercellular adhesion molecule‐1 (*ICAM‐1*) rs5498 polymorphism and ischemic stroke (IS) were investigated by several pilot studies, but with inconsistent results. In this study, a meta‐analysis was performed to better assess the relationship between *ICAM‐1*rs5498 polymorphism and IS.

**Methods:**

PubMed, Medline, Embase, and CNKI were searched for eligible studies. We calculated odds ratios (ORs) and 95% confidence intervals (CIs) to evaluate associations between *ICAM‐1* rs5498 polymorphism and IS.

**Results:**

Totally 16 studies with 2,596 cases and 11,800 controls were analyzed. A significant association with IS was observed for rs5498 polymorphism in GG versus AA + AG (recessive model, *p* = 0.009, OR = 1.62, 95% CI 1.13–2.32, *I* ^2^ = 76%) in overall population. Further subgroup analyses showed that rs5498 polymorphism was significantly associated with IS in Caucasians in AA versus AG + GG (dominant model, *p* = 0.03, OR = 0.52, 95% CI 0.29–0.95, *I* ^2^ = 72%), GG versus AA + AG (recessive model, *p* < 0.0001, OR = 2.98, 95% CI 2.05–4.31, *I* ^2^ = 19%), and A versus G (allele model, *p* = 0.005, OR = 0.50, 95% CI 0.31–0.81, *I* ^2^ = 81%). However, no any positive findings were detected for Asians.

**Conclusions:**

Our findings indicated that rs5498 polymorphism was significantly associated with individual susceptibility to IS in Caucasians, but not in Asians.

## 1 INTRODUCTION

Ischemic stroke (IS) is the leading cause of death and disability worldwide (Global Burden of Disease Study, 2015 Collaborators, 2015). However, despite its high prevalence, the exact cause of IS remains unclear. Recently, accumulating evidence suggests that genetic factors may play crucial parts in its pathogenesis. First, numerous genetic variants were found to be associated with an increased susceptibility to IS by previous genetic association studies (Kopyta, Sarecka‐Hujar, Sordyl, & Sordyl, 2014; Markus & Bevan, 2014; Meschia, Worrall, & Rich, 2011). Second, screening of common causal variants was also proved to be an efficient way to predict the individual risk of developing IS (Chauhan & Debette, 2016). Overall, these findings jointly indicated that genetic predisposition is crucial for the occurrence and development of IS.

Intercellular adhesion molecule‐1 (ICAM‐1) belongs to the immunoglobulin super‐family and plays an important role in regulating inflammatory responses (Hubbard & Rothlein, 2000). It modulates the migration of leukocyte into the tissue and functions as a critical initiator of inflammation (Mousa, 2008). It is well established that excessive inflammation serves as an etiological factor of multiple atherosclerotic/thrombotic vascular diseases including IS (Anrather & Iadecola, 2016; Jin, Liu, Zhang, Nanda, & Li, 2013). Consequently, it is possible that functional *ICAM‐1* polymorphisms, which may affect normal function of ICAM‐1, may also impact individual susceptibility to IS.

Recently, several pilot studies already investigated potential associations between *ICAM‐1*rs5498 polymorphism and IS. However, the results of these studies were inconsistent and the sample size of individual studies was inadequate to draw a definite conclusion (Flex et al., 2004; Li et al., 2009; Motawi, Shaker, Taha, & Abdel Raheem, 2013). In this study, a meta‐analysis was performed to better analyze the role of *ICAM‐1*rs5498 polymorphism in the development of IS.

## 2 MATERIALS AND METHODS

### 2.1 Literature search and inclusion criteria

The current meta‐analysis followed the Preferred Reporting Items for Systematic Reviews and Meta‐analyses guideline (Moher, Liberati, Tetzlaff, Altman, & PRISMA group, 2009). PubMed, Web of Science, Embase, and CNKI were searched for potentially eligible articles using the combination of following terms: “intercellular adhesion molecule‐1”, “ICAM‐1”, “polymorphism”, “variant”, “variation”, “mutation”, “genotype”, “allele”, “ischemic stroke”, “cerebral infarction”, “brain infarction”, “cerebral ischemia”, “brain ischemia”, “transient ischemic attack”, “cerebrovascular disease”, “IS”, “CI”, “TIA”, and “CVD”. Additionally, the reference lists of all retrieved articles were also screened.

To test the research hypothesis of this meta‐analysis, included studies should meet all the following criteria: (a) case–control study about *ICAM‐1* rs5498 polymorphism and IS; (b) providing sufficient data for calculating odds ratios (ORs) and 95% CIs; (c) full text in English or Chinese available. Studies were excluded if one of the following conditions was fulfilled: (a) not related to *ICAM‐1* rs5498 polymorphism and IS; (b) pedigree studies; (c) case reports or case series. In the case of duplicate reports by the same authors, we only included the most complete study.

### 2.2 Data extraction and quality assessment

We extracted the following information from eligible studies: (a) name of the first author; (b) year of publication; (c) country and ethnicity of participants; (d) type of disease; (e) sample size; and (f) the genotypic distribution of *ICAM‐1* rs5498 polymorphism in cases and controls. The probability value (*p* value) of Hardy–Weinberg equilibrium (HWE) was also calculated.

We used the Newcastle‐Ottawa scale (NOS) to evaluate the quality of eligible studies (Stang, 2010). The NOS has a score range of zero to nine, and studies with a score of more than seven were thought to be of high quality.

Two reviewers conducted data extraction and quality assessment independently. When necessary, we wrote to the corresponding authors for extra information. Any disagreement between two reviewers was solved by discussion until a consensus was reached.

### 2.3 Statistical analyses

In the current meta‐analysis, we performed statistical analyses by using Review Manager Version 5.3.3. We calculated ORs and 95% CIs to estimate potential associations between *ICAM‐1* rs5498 polymorphism and IS in dominant (AA vs. AG + GG), recessive (GG vs. AA + AG), over‐dominant (AG vs. AA + GG) and allele (A vs. G) models, and a *p* value of 0.05 or less was defined as statistically significant. Between‐study heterogeneities were evaluated by *I* ^2^ statistic. Random‐effect models would be used for analyses if *I* ^2^ was greater than 50%. Otherwise, analyses would be conducted with fixed‐effect models. Subgroup analyses were subsequently carried out by ethnicity of participants and type of disease. Stabilities of synthetic results were tested in sensitivity analyses. Publication biases were assessed by funnel plots.

## 3 RESULTS

### 3.1 Characteristics of included studies

**Figure 1:**
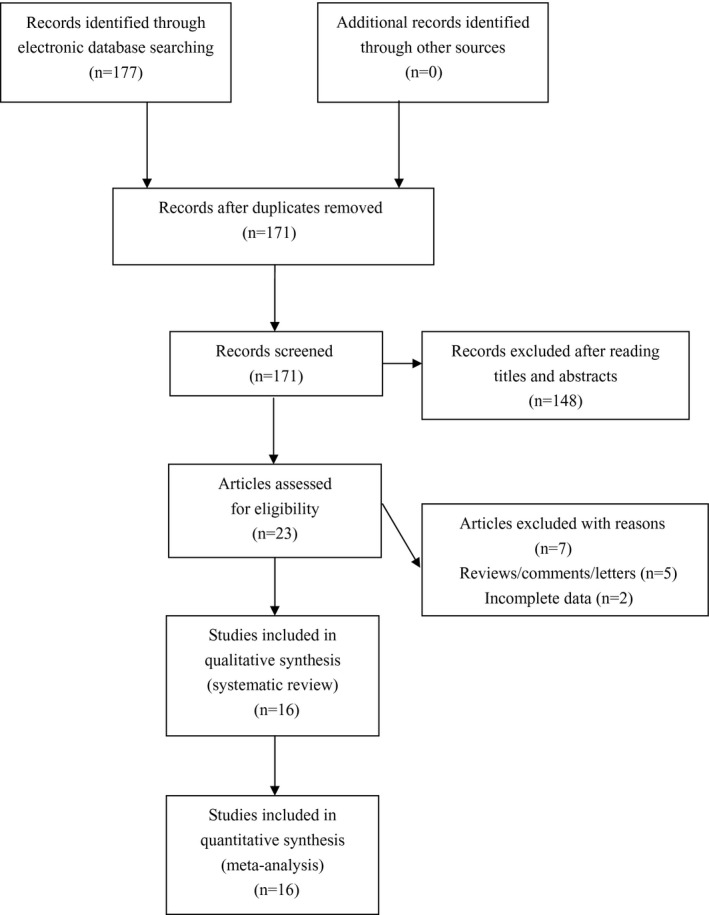
Flowchart of study selection for the present study

The initial literature search identified 177 articles. After excluding irrelevant and duplicate articles, 23 articles were retrieved for further evaluation. Another seven articles were subsequently excluded after reading the full text. Ultimately, a total of 16 eligible studies involving 2,596 cases and 11,800 controls were enrolled for analyses (see Figure 1). Characteristics of included studies are summarized in Table 1.

**Table 1:** The characteristics of included studies for *ICAM‐1* rs5498 polymorphism and IS

First author, year	Country	Ethnicity	Type of disease	Sample size	Genotype distribution		*p*‐Value for HWE	NOS score
					Cases	Controls		
Flex et al., 2004	Italy	Caucasian	IS	237/223	72/112/53	75/125/23	0.006	8
Geng, 2016	China	Asian	CI	180/180	119/39/22	85/72/23	0.216	8
Gu, 2012	China	Asian	CI	120/102	28/52/40	39/46/17	0.585	8
Guo, 2011	China	Asian	CI	115/99	35/60/20	50/42/7	0.649	7
Li, 2009	China	Asian	IS	309/309	148/132/29	192/102/15	0.760	8
Liu, 2005	China	Asian	CI	142/101	65/65/12	50/44/7	0.519	7
Lu, 2013	Taiwan	Asian	IS	312/332	196/100/16	250/80/2	0.099	8
Motawi et al., 2013	Egypt	Caucasian	IS	63/75	21/15/27	45/21/9	0.018	8
Pola, 2003	Italy	Caucasian	IS	119/133	24/63/32	49/68/16	0.301	8
Shang, 2004	China	Asian	CI	53/71	25/17/11	42/24/5	0.545	8
Sun, 2005	China	Asian	CI	112/105	59/45/8	42/44/19	0.220	7
Volcik, 2010	USA	Mixed	IS	290/9,593	99/138/53	3,111/4,836/1646	0.002	7
Wang, 2015	China	Asian	IS	50/50	41/4/5	35/7/8	<0.001	7
Wei, 2005	China	Asian	IS	205/210	74/88/32	95/97/18	0.329	8
Xing, 2006	China	Asian	CI	112/105	59/35/18	29/44/32	0.099	7
You, 2006	China	Asian	IS	177/112	72/86/19	62/40/10	0.343	8

IS: Ischemic stroke; CI: Cerebral infarction; HWE: Hardy–Weinberg equilibrium; NOS: Newcastle‐Ottawa scale; ICAM‐1: intercellular adhesion molecule‐1.

### 3.2 Overall and subgroup analyses

A significant association with IS was observed for rs5498 polymorphism in GG versus AA + AG (recessive model, *p* = 0.009, OR = 1.62, 95% CI 1.13–2.32, *I* ^2^ = 76%) in overall population. Further subgroup analyses showed that rs5498 polymorphism was significantly associated with IS in Caucasians in AA versus AG + GG (dominant model, *p* = 0.03, OR = 0.52, 95% CI 0.29–0.95, *I* ^2^ = 72%), GG versus AA + AG (recessive model, *p* < 0.0001, OR = 2.98, 95% CI 2.05–4.31, *I* ^2^ = 19%), and A versus G (allele model, *p* = 0.005, OR = 0.50, 95% CI 0.31–0.81, *I* ^2^ = 81%). However, no any positive findings were detected for Asians (see Table 2 and Figure s1).

**Table 2:** Results of overall and subgroup analyses for ICAM‐1 rs5498 polymorphism and IS

Population	Sample size	Dominant comparison			Recessive comparison			Overdominant comparison			Allele comparison		
		*p* value	OR (95% CI)	*I* ^2^ statistic	*p* value	OR (95% CI)	*I* ^2^ statistic	*p* value	OR (95% CI)	*I* ^2^ statistic	*p* value	OR (95% CI)	*I* ^2^ statistic
Overall	2,596/11,800	0.14	0.81 (0.61–1.07)	83%	**0.009**	**1.62 (1.13–2.32)**	76%	0.75	0.97 (0.80–1.17)	63%	0.05	0.79 (0.62–1.00)	87%
Asian	1,887/1,776	0.48	0.88 (0.61–1.26)	85%	0.15	1.40 (0.88–2.21)	74%	0.96	1.01 (0.78–1.30)	69%	0.35	0.86 (0.64–1.17)	88%
Caucasian	419/431	**0.03**	**0.52 (0.29–0.95)**	72%	**<0.0001**	**2.98 (2.05–4.31)**	19%	0.15	0.82 (0.62–1.07)	0%	**0.005**	**0.50 (0.31–0.81)**	81%
CI	834/763	0.89	0.96 (0.55–1.69)	87%	0.44	1.12 (0.85–1.47)	79%	0.33	0.85 (0.61–1.18)	61%	0.94	0.98 (0.61–1.57)	90%

Note

The values in bold represent that there is statistically significant differences between cases and controls.

CI: Cerebral infarction; OR: Odds ratio; CI: Confidence interval; ICAM‐1: intercellular adhesion molecule‐1; IS: Ischemic stroke.

### 3.3 Sensitivity analyses

We conducted sensitivity analyses by eliminating one individual study each time. No any alterations of results were detected in sensitivity analyses, which suggested that our findings were statistically stable.

### 3.4 Publication biases

We used funnel plots to evaluate potential publication biases. The shape of funnel plots was symmetry for every comparison, which indicated that severe publication biases were unlikely.

## 4 DISCUSSION

As far as we know, this is so far the most comprehensive meta‐analysis about *ICAM‐1* rs5498 polymorphism and IS. The pooled analyses revealed that *ICAM‐1* rs5498 polymorphism was significantly associated with individual susceptibility to IS in Caucasians, but not in Asians. The stabilities of synthetic results were evaluated by sensitivity analyses, and no alterations of results were observed in any comparisons, which suggested that our findings were statistically stable.

There are several points that worth noting about this meta‐analysis. First, the rs5498 polymorphism causes an amino acid change from glutamic acid (E) to lysine (K), which is proved to be associated with alteration in protein structure and binding affinity of ICAM‐1 protein (He et al., 2014; Mohamed et al., 2010). Thus, it is possible that rs5498 polymorphism may impact biological function of ICAM‐1, give rise to the development of over‐activated inflammatory reactions, and ultimately influence individual susceptibility to IS. Second, the etiology of IS is extremely complex, and as a consequence, to better elucidate potential roles of genetic variations in IS, we strongly recommend future studies to conduct haplotype analyses and investigate potential gene‐gene interactions (Xie, Shi, & Liu, 2017). Third, it is noteworthy that the sample sizes of pooled analyses in this meta‐analysis were still relatively small, so further studies are still needed to test the associations between rs5498 polymorphism and IS, especially in Caucasians.

Some limitations of this meta‐analysis should also be noted when interpreting our findings. First, our pooled analyses were based on unadjusted estimations due to the lack of raw data, and we have to admit that failure to perform further adjusted analyses may impact the reliability of our findings (Shi, Xie, Jia, & Li, 2016). Second, since our pooled analyses were based on case–control studies, despite our positive findings, future perspective studies are still needed to examine whether there is direct causal relationship between rs5498 polymorphism and IS (Shi et al., 2015). Third, associations between rs5498 polymorphism and IS may also be modified by gene‐environmental interactions. However, most studies did not consider these potential interactions, which impeded us to conduct relevant analyses (Liu, Wu, & Liu, 2013). Fourth, gray literatures like abstracts and other research materials that were not formally published in academic journals were not considered to be eligible for analyses in this meta‐analysis since it is hard to determine their quality. However, since gray literatures were not analyzed, although funnel plots suggested that severe publication biases were unlikely, it is still possible that our findings may be impacted by potential publication biases (Song & Lee, 2015). Considering the above mentioned limitations, our findings should be interpreted with caution.

In summary, our meta‐analysis suggested that *ICAM‐1* rs5498 polymorphism might serve as a genetic biomarker of IS in Caucasians, but not in Asians. However, further well‐designed studies, especially in Caucasian are still warranted to confirm our findings.

## ETHICAL APPROVAL

This article does not contain any studies with human participants or animals performed by any of the authors.

## CONFLICT OF INTEREST

The authors declare that they have no conflict of interest.

## AUTHORS' CONTRIBUTIONS

Hua Gao conceived of the study, participated in its design. Hua Gao and Xinhua Zhang conducted the systematic literature review. Hua Gao and Xinhua Zhang performed data analyses. Hua Gao drafted the manuscript. All authors have read and approved the final manuscript.
