# Supplementary material for: Associations of intercellular adhesion molecule‐1 rs5498 polymorphism with ischemic stroke: A meta‐analysis
Source: Mol Genet Genomic Med. 2019 Apr 17;7(6):e643. doi: 10.1002/mgg3.643 (PMC6565590; doi:10.1002/mgg3.643)
Supplement: Supplementary file 1 [file MGG3-7-e643-s001.docx]

**Forest plots of investigated polymorphisms**


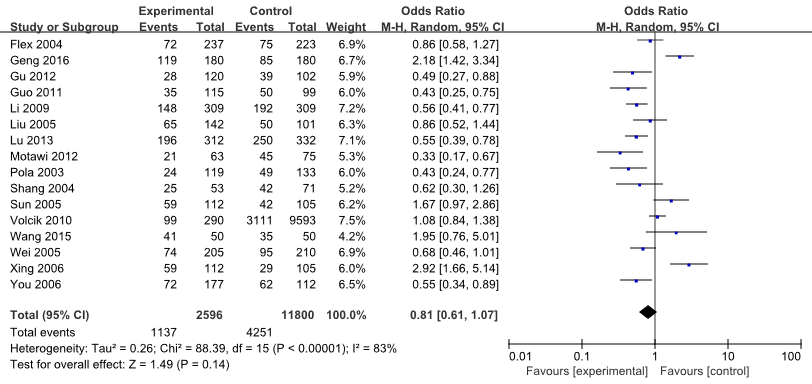


Forest plot of the rs5498 polymorphism and IS under dominant comparison.


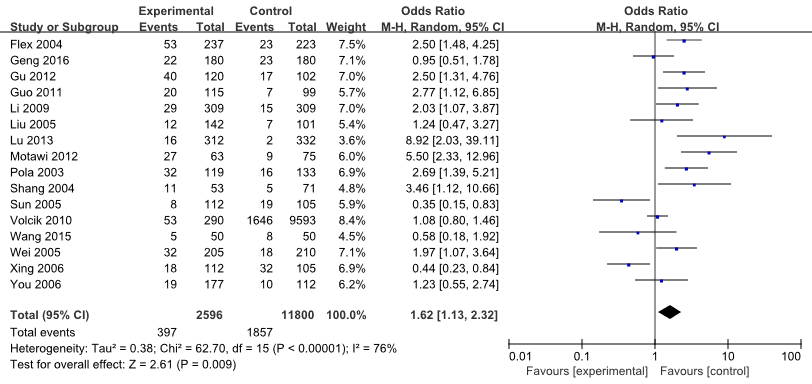


Forest plot of the rs5498 polymorphism and IS under recessive comparison.


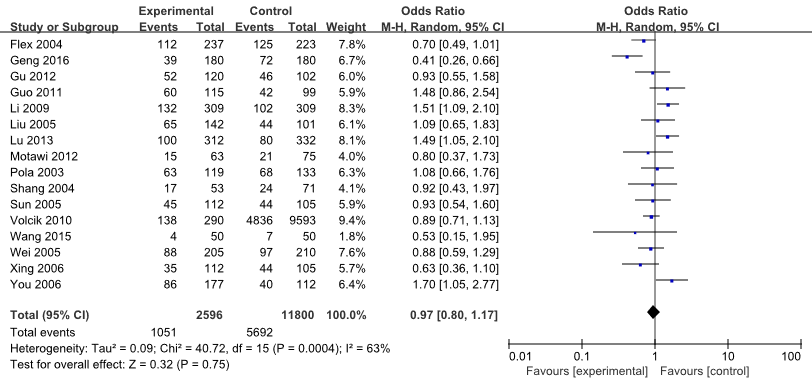


Forest plot of the rs5498 polymorphism and IS under additive comparison.


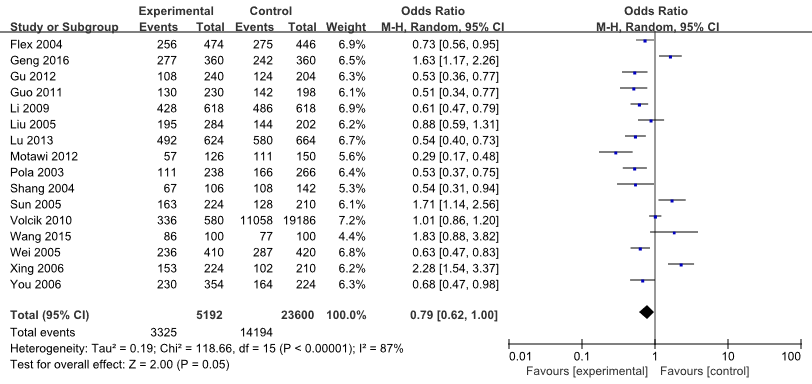


Forest plot of the rs5498 polymorphism and IS under allele comparison.
